# MicroRNA function is required for neurite outgrowth of mature neurons in the mouse postnatal cerebral cortex

**DOI:** 10.3389/fncel.2013.00151

**Published:** 2013-09-13

**Authors:** Janet Hong, Haijun Zhang, Yoko Kawase-Koga, Tao Sun

**Affiliations:** ^1^Department of Cell and Developmental Biology, Cornell University Weill Medical CollegeNew York, NY, USA; ^2^Department of Oral and Maxillofacial Surgery, The University of Tokyo HospitalTokyo, Japan

**Keywords:** miRNAs, Dicer, neurogenesis, neurite outgrowth, cerebral cortex

## Abstract

The structure of the postnatal mammalian cerebral cortex is an assembly of numerous mature neurons that exhibit proper neurite outgrowth and axonal and dendritic morphology. While many protein coding genes are shown to be involved in neuronal maturation, the role of microRNAs (miRNAs) in this process is also becoming evident. We here report that blocking miRNA biogenesis in differentiated neurons results in microcephaly like phenotypes in the postnatal mouse brain. The smaller brain defect is not caused by defective neurogenesis, altered neuronal migration or significant neuronal cell death. Surprisingly, a dramatic increase in neuronal packing density within the postnatal brain is observed. Loss of miRNA function causes shorter neurite outgrowth and smaller soma size of mature neurons *in vitro*. Our results reveal the impact of miRNAs on normal development of neuronal morphology and brain function. Because neurite outgrowth is critical for neuroregeneration, our studies further highlight the importance of miRNAs in the treatment of neurological diseases.

## INTRODUCTION

In the mammalian cerebral cortex, projection neurons are generated from radial glial cells (RGCs) and intermediate progenitors (IPs) that reside in the ventricular zone (VZ) and subventricular zone (SVZ), respectively (Noctor et al., 2001; Rakic, 2003; Haubensak et al., 2004; Englund et al., 2005; Gotz and Huttner, 2005). Postmitotic neurons (PNs) differentiate and migrate into the cortical plate (CP), in which PNs are organized in an inside–out six layered structure, with earliest born neurons in the deep layers and later born neurons in the upper layers (Guillemot, 2005; Molyneaux et al., 2007). Proper neurite outgrowth and axonal and dendritic morphogenesis are critical for neuronal maturation, synaptic formation, and neuronal function (Frank and Tsai, 2009; Merot et al., 2009). Molecular mechanisms regulating neuronal differentiation and maturation remain an exciting research topic.

The importance of microRNAs (miRNAs)-mediated neurogenesis and neuronal maturation in the central nervous system (CNS) has drawn significant attention (Kosik, 2006; Fineberg et al., 2009; Shi et al., 2010; Bian and Sun, 2011). MiRNAs are approximately 22 nucleotide (nt) endogenous non-coding small RNAs (Lee et al., 1993; Wightman et al., 1993). A mature miRNA recognizes a complementary sequence in the 3′-untranslated region (3′-UTR) of its target messenger RNA (mRNA) and affects mRNA stability and/or silences protein translation (Carthew and Sontheimer, 2009; Kim et al., 2009). Because miRNA precursors are processed into mature miRNAs by the RNAase III enzyme Dicer, the role of miRNAs in neurogenesis has been demonstrated by regional-specific deletion of *Dicer* expression in the CNS using different *Cre* lines (Volvert et al., 2012; Zhang et al., 2012). For example, *Dicer* ablated knockout (Ko) mice in PNs using the *CaMKII-Cre* line display impaired dendritic branching in pyramidal neurons in the CA1 region of the hippocampus (Davis et al., 2008; Hebert et al., 2010). These studies indicate the importance of miRNA functions in morphogenesis of mature neurons in the brain.

In this study, we demonstrate the critical role of miRNAs in neurite outgrowth of mature cortical neurons. Blocking miRNA biogenesis in PNs in the mouse cortex at perinatal stages does not significantly affect neurogenesis, neuronal survival, and layer organization. However, the neuronal packing density is greatly increased in the CP, resulting in a significantly reduced cortical size. Correspondingly, neurite outgrowth and soma size development are significantly reduced in cultured *Dicer* Ko PNs. Our results demonstrate that miRNA functions are required for proper neuronal maturation. Moreover, our studies suggest a potential role of miRNAs in promoting neurite outgrowth in the treatment of neurodegenerative diseases.

## MATERIALS AND METHODS

### GENERATION OF *Dicer* CONDITIONAL KNOCKOUT MICE

The floxed Dicer transgenic mice (*Dicer*^flox/flox^; C57/BL6 × 129 background; kindly provided by the Greg Hannon’s lab at the Cold Spring Harbor Laboratory; Murchison et al., 2005) were bred with *Nex-Cre* mice (C57/BL6 background, provided by Drs M. Schwab and K. Nave at Max-Planck-Institute of Experimental Medicine, Goettingen, Germany; Goebbels et al., 2006) to generate *Nex-Cre-Dicer* Ko (*Nex-Cre*; *Dicer*^flox/flox^) animals.

For staging of embryos, mid-day of the day of vaginal plug formation is considered embryonic day 0.5 (E0.5), and the first 24 h after birth is defined as postnatal day 0 (P0). Animal use was overseen by the Animal Facility at the Weill Cornell Medical College.

### GENOTYPING OF *Dicer* CONDITIONAL KNOCKOUT MICE

Mouse tail tip biopsies were used for genotyping by polymerase chain reaction reactions using the following primer pairs: for *Cre*, 5′-TAAAGATATCTCACGTACTGACGGTG-3′ and 5′-TCTCTGACCAGAGTCATCCTTAGC-3′ (product size: 350 bp); for *Dicer*, 5′-ATTGTTACCAGCGCTTAGAATTCC-3′ and 5′-GTACGTCTACAATTGTCTATG-3′ (product sizes: 767 bp from the floxed *Dicer* allele and 560 bp from the wild-type *Dicer* gene).

### BREEDING THE *Nex-Cre* LINE WITH FLOXED LacZ REPORTER MICE

To localize the *Cre* activity sites, *Nex-Cre* transgenic mice were crossed with homozygous ROSA26 floxed LacZ reporter mice, obtained from Jackson Laboratories (Bar Harbor, Maine). The ROSA26 mice carry a loxP-flanked transcriptional “STOP” DNA sequence that prevents the transcription of the *LacZ* gene. Only the cells that express the *Cre* recombinase can remove the “STOP” sequence and subsequently activate the transcription of the *LacZ* gene. Cells which express LacZ produce a blue color in the β-galactosidase assay (X-gal staining).

### β-GALACTOSIDASE ACTIVITY ASSAY

Mouse brains were dissected in ice-cold 1× phosphate buffered saline (PBS) and placed in 4% paraformaldehyde (PFA) in PBS for 15 min at room temperature. Fixed brains were washed in PBS for 3 × 5 min and sectioned coronally (100 μm) using a Leica vibratome (Leica, VT1000 S). Brain sections were washed three times in a wash solution (0.1 M phosphate buffer and 2 mM MgCl_2_) and subjected to a 5-bromo-4-chloro-3-indolyl-β-D-galactopyranoside (X-gal) solution (1 mg/ml X-gal and 5 mm potassium ferrocyanide, 5 mm potassium ferricyanide in wash buffer) for 30 min to 1 h at 37° C. The reaction was quenched by washing sections three times in wash solution and incubating them in 4% PFA in PBS for 5 min at room temperature. The sections were washed three times in wash solution and mounted with a coverslip. The images were collected using a Leica digital camera under a dissection scope (Leica, MZ16F).

### TISSUE PREPARATION AND IMMUNOHISTOCHEMISTRY

Mouse brains were collected and fixed in 4% PFA in PBS at 4° C overnight, followed by incubating in 30% sucrose in PBS. Brain tissues were embedded in optimal cutting temperature (OCT) and stored at -80° C until use. Brains were sectioned coronally (14 μm) using a Leica cryostat (Leica, CM3050 S).

For antigen recovery, sections were incubated in heated (95–100° C) antigen recovery solution [1 mM ethylenediaminetetraacetic acid (EDTA), 5 mM Tris, pH 8.0] for 15–20 min, and cooled down for 20–30 min. Before applying antibodies, sections were blocked in 10% normal goat serum (NGS) in PBS with 0.1% Tween-20 (PBT) for 1 h. Sections were incubated with primary antibodies at 4° C overnight and visualized using goat anti-rabbit IgG–Alexa-Fluor-488 and/or goat anti-mouse IgG–Alexa-Fluor-546 (1:350, Molecular Probes) for 1.5 h at room temperature. Images were captured using a Leica digital camera under a fluorescent microscope (Leica DMI6000B).

Primary antibodies against the following antigens were used: bromodeoxyuridine (BrdU; 1:50, DSHB), Ki67 (1:500, Abcam), Tbr1 (1:2500, Abcam), Ctip2 (1:1000, Abcam), Cux1 (1:200, Santa Cruz), Satb2 (1:1000, Abcam), β-tubulin III (TuJ1; 1:500, Chemicon), Map2 (1:500, Chemicon), and NeuN (1:300, Chemicon).

### NISSL STAINING

Sections (14 μm) were processed through incubation in the following solutions in order: ethanol/chloroform (1:1, overnight), 100% ethanol (30 s), 95% ethanol (30 s), distilled water (30 s, twice), cresyl violet (3–5 min), distilled water (2 min, three times), 50% ethanol (2 min), 95% ethanol (5–30 min), 100% ethanol (5 min, twice), xylene (3 min, twice), and then mounted with a coverslip. The images were collected using a Leica digital camera under a dissection scope (Leica, MZ16F).

### *IN SITU* HYBRIDIZATION

*In situ* hybridization for miRNA expression was performed according to previously published methods with modifications using locked nucleic acid (LNA) probes (Obernosterer et al., 2007). Briefly, after fixation with 4% PFA, acetylation with acetylation buffer (13.33% triethanolamince, 2.5% acetic anhydride, 20 mM HCl), treatment of proteinase K (10 mg/ml, IBI Scientific) and pre-hybridization [1× saline-sodium citrate (SSC), 50% formamide, 0.1 mg/ml salmon sperm DNA solution, 1× Denhardt, 5 mM EDTA, pH 7.5], brain sections were hybridized with digoxigenin (DIG)-labeled LNA probes at a proper temperature (Tm-22° C) overnight. After washing with pre-cooled wash buffer (1× SSC, 50% formamide, 0.1% Tween-20) and 1× maleic acid buffer containing Tween 20 (MABT), sections were blocked with blocking buffer (1× MABT, 2% blocking solution, 20% heat-inactived sheep serum) and incubated with anti-DIG antibody (1:1,500, Roche) at 4° C overnight. Brain sections were washed with 1× MABT and staining buffer (0.1M NaCl, 50 mM MgCl_2_, 0.1M Tris–HCl, pH 9.5), stained with BM purple (Roche) at room temperature until ideal intensity. The microRNA LNA probes (Exiqon) were 3′ end labeled with DIG–ddUTP with terminal transferase using the DIG–3′ end labeling kit (Roche).

The images of *in situ* hybridization were collected using a Leica digital camera under a dissection scope (Leica, MZ16F).

### BrdU INCORPORATION

To assess proliferation of neural progenitors in the developing cortex, one dose of BrdU (50 μg/g body weight) was administrated by intraperitoneal injection to timed-pregnant female mice half an hour before sacrifice.

### TUNEL ASSAY

To identify apoptotic cells in the cortex, we performed a TUNEL (terminal deoxynucleotidyl transferase dUTP nick end labeling) assay using an Apop Tag Fluorescein *in situ* Apoptosis detection kit (Chemicon) on 14-μm frozen sections. This assay was performed according to the manufacturer’s instructions.

### CELL COUNTING IN THE CORTICAL WALL

Coronal sections were collected in the medial cortical region (at levels between the anterior commissure and the anterior hippocampus). At least four sections from each brain and three brains from different litters were chosen for antibody labeling and TUNEL assay. For **Figures 2** and **3**, positive cells were quantified in fixed areas of 186 μm × 1200 μm in the cortical wall of P5 and P10 cortices. For **Figure 4**, positive cells were quantified in fixed areas of 186 μm × 186 μm in the cortical wall of P5 and P10 cortices.

### PRIMARY NEURONAL CULTURES

Neuronal cultures were performed according to established protocols (Yu et al., 2005) with modifications. Briefly, the dorsal cortex was dissected from the P0 brain, and transferred to pre-cooled Hanks’ balanced salt solution (HBSS) medium. Tissue was dissociated with 0.5 mg/ml DNAse I (Sigma D4527) in HBSS for 2 min at 37° C and mechanically triturated with fire-polished Pasteur pipettes into a single cell suspension. Cortical neurons were plated onto poly-L-lysine (PLL) and Laminin treated coverslips at 5 × 10^4^ cells/well in 24-well plates. Neuronal cultures were maintained in neuronal medium [Dulbecco’s modified Eagle medium (DMEM)/F12, N2, B27, glucose, NaHCO_3_, HEPES (4-(2-hydroxyethyl)-1-piperazineethanesulfonic acid)] with fibroblast growth factor 2 (FGF-2; 20 ng/ml; Invitrogen) treatment for the first 24 h only. Afterward, cells were cultured in neuronal medium only and medium was changed every 2–3 days.

Primary neurons were fixed after 10 days *in vitro* (DIV 10) with 4% PFA in PBS for 30 min at room temperature. Before applying antibodies, cells were blocked in 10% NGS in PBS with 0.3% Triton X-100 for 1 h. Cells were incubated with primary antibodies at 4° C overnight and visualized using goat anti-rabbit IgG–Alexa-Fluor-488 and/or goat anti-mouse IgG–Alexa-Fluor-546 (1:350, Molecular Probes) for 1.5 h at room temperature. Images were captured using a Leica digital camera under a fluorescent microscope (Leica DMI6000B).

### ANALYSIS OF NEURITE GROWTH AND SOMA SIZE

Typically, pictures of 30–50 neurons from three separate coverslips from each experiment were taken using a Leica digital camera under a fluorescent microscope (Leica DMI6000B). Representative cells with strong Map2 and Tuj1 immunoreactivity labeling neurite (axonal and dendritic) processes were analyzed. Neurites that had lengths that were at least twice the diameter of the cell body were measured. Neurite lengths from the soma and soma size area were traced and measured using Image J software and the data were compiled and analyzed using the Excel program (Microsoft).

### STATISTIC ANALYSIS

At least three *Nex-Cre-Dicer Ko* (*Ko*) and three control (*Ctrl*) animals were used for all statistical analyses. Data were shown as mean ± SEM. Statistical comparison was made by analysis of variance (unpaired *t*-test or analyses of variance). Additional details regarding the *n* (number of animals) or *N* (number of neurites or cells) are found in the pertinent figure legend.

## RESULTS

### CORTICAL GROWTH DEFECTS IN *Nex-Cre-Dicer* KNOCKOUT MICE

**FIGURE 1:**
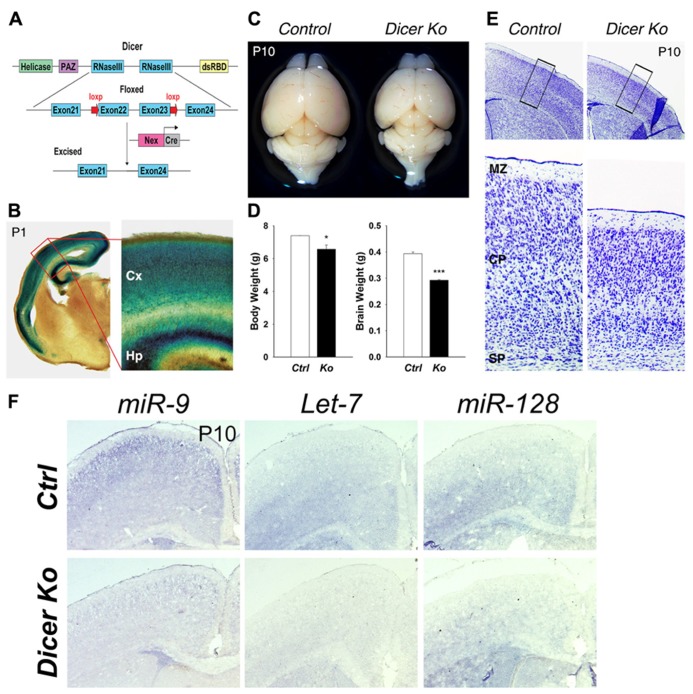
**Conditional ablation of Dicer in postmitotic neurons of the cerebral cortex results in a smaller cortex and reduced thickness of the cortical wall.** **(A)** Dicer targeting construct. The N-terminal RNA helicase domain, piwi argonaute and zwille (PAZ) domain, two ribonuclease III domains, and a double-stranded RNA- binding domain (RBD) are labeled. The exon 22 and exon 23 of Dicer are conditionally excised after *Nex-Cre* recombination. **(B)** X-gal staining at the level of the cortex in a *Nex-Cre* and *Rosa26-LacZ*- expressing mouse at P1 illustrating cortical specificity of the *Nex-Cre* line. The red box indicates the region shown at higher magnification. The cortex (Cx) and hippocampus (Hp) are labeled. **(C)** Appearance of representative brains from P10 control and *Nex-Cre-Dicer Ko* mice (litter mates). **(D)** Body and brain weights of control (*Ctrl*) and *Nex-Cre-Dicer Ko* (*Ko*) mice at P10. **(E)** Coronal sections of P10 brains with Nissl staining of control and *Nex-Cre-Dicer Ko* mice. The black boxes indicate the region shown at higher magnification. The subplate (SP), cortical plate (CP), and marginal zone (MZ) are labeled. **(F)** *In situ* hybridization of *miR-9*, *Let-7*, and *miR-128* in control and *Dicer Ko* cortices at P10. Data are presented as mean ± SEM; *n* ≥ 3 in all genotypes; *p* values in relation to control (*p < 0.05, ***p < 0.00002).

To examine the role of miRNAs in the maturation of differentiated neurons, we conditionally ablated Dicer expression in PNs in the mouse cerebral cortex utilizing a *Cre*-loxp system. A floxed Dicer mouse line (*Dicer*^flox/flox^) with two loxP sites flanking exon 22 and exon 23, which encode the RNAase III domains of *Dicer*, were bred with a *Nex-Cre* mouse line to generate *Nex-Cre-Dicer Ko* mice (**Figure 1A**). The *Nex-Cre* line displays activity by E13.5 and is prominently expressed in differentiating neurons of the dorsal telencephalon without affecting proliferating precursor cells of the VZ (Goebbels et al., 2006). Proliferating precursor cells can be detected by labeling cells in the S phase with a 30 min pulse of BrdU, and in the G1, S, G2, and M phase with Ki67. Indeed, quantification of BrdU^+^ and Ki67^+^ cells revealed no change in E15.5 *Nex-Cre-Dicer Ko* cortices compared to controls (data not shown). As such, Dicer and consequently miRNA production was conditionally ablated in PNs in the cortex after *Cre* recombination, as demonstrated by X-gal staining in P1 cortices of mice bred between the *Nex-Cre* line and the *Rosa26-LacZ* reporter line (**Figure 1B**).

Inactivation of Dicer in differentiated neurons caused markedly reduced postnatal growth. Moreover, *Nex-Cre-Dicer Ko* mice could not survive past P23, presumably due to starvation and dehydration after weaning. At P1, the brain size of *Nex-Cre-Dicer Ko* mice was comparable to that of controls (data not shown). However, gross brain morphology at P10 revealed a significant size reduction in *Nex-Cre-Dicer Ko* brains compared to controls (**Figure 1C**). Quantification of the body and brain weights of P10 *Dicer Ko* mice showed a significant reduction compared to controls, with a more profound reduction in brain weight (**Figure 1D**). Next, cortical morphology was analyzed in coronal sections of P10 brains by Nissl staining. While overall cortical lamination appeared normal, the thickness of the cortical wall was significantly reduced in *Nex-Cre-Dicer Ko* brains compared to controls (**Figure 1E**).

To verify that the brain phenotypes were caused by miRNA loss, we performed miRNA *in situ* hybridization in control and *Dicer Ko* brains. Three brain-enriched miRNAs, miR-9, Let-7, and miR-128, were utilized. We found that expression levels of all three miRNAs were reduced in P1 cortices and almost diminished in P10 cortices, suggesting a progressive loss of miRNAs due to Dicer deletion (**Figure 1F** and data not shown).

Our results indicate that Dicer deletion in differentiated neurons in the developing brain causes early postnatal death, reduced body and brain weights, and severe reduction of the cortical wall.

### DEPLETION OF miRNA FUNCTION IN POSTMITOTIC NEURONS DOES NOT SIGNIFICANTLY AFFECT CORTICAL LAMINATION AND NEURONAL PRODUCTION

**FIGURE 2:**
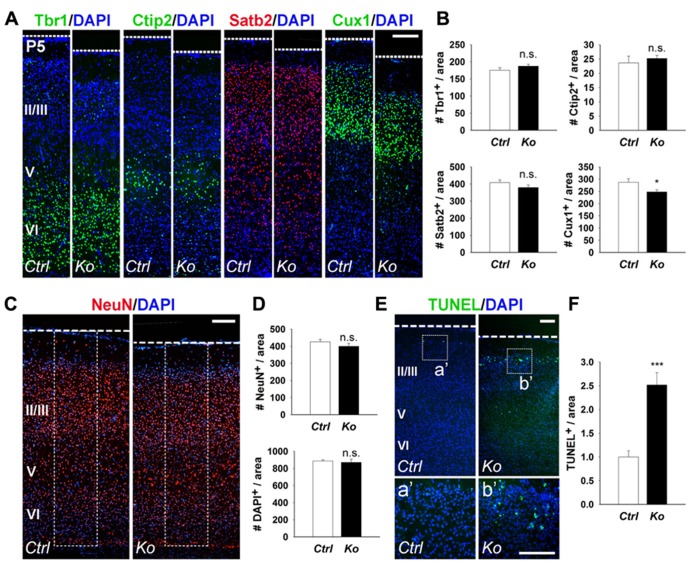
**Conditional Dicer loss in mature neurons does not significantly alter cortical lamination and neuronal production, and exhibits a transitory increase in cell death at P5.** **(A,B)** The numbers of early-born neurons labeled with Tbr1 and Ctip2 were unaffected in P5 *Nex-Cre-Dicer Ko* (*Ko*) cortices, compared to controls (*Ctrl*). The numbers of late-born neurons labeled with Satb2 were unaffected and Cux1 were decreased in P5 *Dicer Ko* cortices, compared to controls. **(C,D)** Numbers of NeuN^+^ and DAPI^+^ cells were unaffected in the cortical wall of P5 *Dicer Ko* cortices compared to controls. The dashed box indicates the area of quantification. **(E)** TUNEL assay of coronal cryosections of P5 control and *Dicer Ko* cortices. The dashed box indicates the region shown at higher magnification in panel **a**′, **b**′. **(F)** Normalized quantification of TUNEL^+^ cells per area in the cortical wall of P5 control and *Dicer Ko* brains. Cortical layers (VI), (V), and (II/III) are labeled. Scale bar: 100 μm. Data are presented as mean ± SEM; *n* ≥ 3 in all genotypes; *p* values in relation to control (*p < 0.04, ***p < 0.0002). n.s., not significant.

Due to the significant reduction in cortical wall thickness, we investigated the effects of Dicer ablation on the generation of early- and late-born neurons. During mouse cortical development, early-born neurons generated at E12.5–E13.5 migrate and form deep cortical layers VI and V, and express Tbr1 and Ctip2, respectively (Arlotta et al., 2005; Kolk et al., 2006; Chen et al., 2008; Han et al., 2011). Late-born neurons are generated at E14.5–E18.5 and migrate to form the upper neuronal layers II–IV above the deep cortical layers, and can be detected by Satb2 and Cux1 expression (Alcamo et al., 2008; Britanova et al., 2008; Cubelos et al., 2010). We first examined early- and late-born neuron production in the P5 cortex. Quantification of early-born neurons with Tbr1^+^ and Ctip2^+^ cells revealed no significant difference between control and *Nex-Cre-Dicer Ko* cortices (**Figures 2A,B**). For late-born neurons, Satb2^+^ cells were unaffected but Cux1^+^ cells were slightly decreased in *Dicer Ko* cortices compared to controls (**Figures 2A,B**). Next, we analyzed neuronal production in the P5 cortex. Quantification of neuron and cell numbers by NeuN and DAPI immunostaining showed no significant difference in *Nex-Cre-Dicer Ko* cortices compared to controls (**Figures 2C,D**).

Given that Dicer ablation did not reveal a significant defect in cortical lamination and neuronal production despite the reduced cortical thickness, we investigated the possibility of neuronal cell death. Apoptotic cells in the cortex were detected by TUNEL assay. At P5, there was a significant increase in apoptotic cells in *Nex-Cre-Dicer Ko* cortices compared to controls, which was not detected in P1 cortices (**Figures 2E,F** and data not shown). Moreover, TUNEL^+^ cells in *Dicer Ko* brains were localized in the far-upper cortical layer at the marginal zone boundary, suggesting apoptosis of a subset of late-born neurons (**Figures 2Ea′,b′**).

**FIGURE 3:**
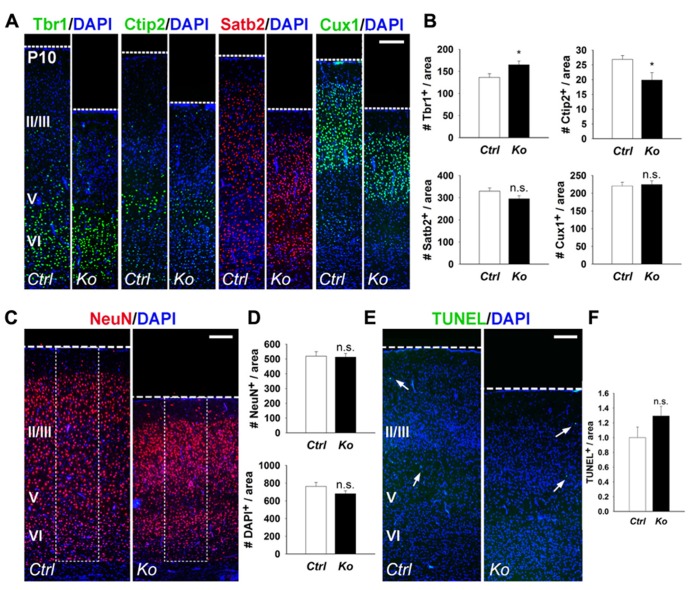
**Depletion of miRNA function in postmitotic neurons slightly alters early-born neurons and has no measured effect on neuronal production and cell death at P10. (A,B)** The numbers of early-born neurons labeled with Tbr1 were increased and Ctip2 were decreased in P10 *Nex-Cre-Dicer Ko* (*Ko*) cortices, compared to controls (*Ctrl*). The numbers of late-born neurons labeled with Satb2 and Cux1 were unaffected in P10 *Dicer Ko* cortices, compared to controls. **(C,D)** Numbers of NeuN^+^ and DAPI^+^ cells were unaffected in the cortical wall of P10 *Dicer Ko* cortices compared to controls. The dashed box indicates the area of quantification. **(E)** TUNEL assay of coronal cryosections of P10 control and *Dicer Ko* cortices. Arrows indicate TUNEL^+^ cells. **(F)** Normalized quantification of TUNEL^+^ cells per area in the cortical wall of P10 control and *Dicer Ko* brains. Cortical layers (VI), (V), and (II/III) are labeled. Scale bar: 100 μm. Data are presented as mean ± SEM; *n* ≥ 3 in all genotypes; *p* values in relation to control (*p < 0.04). n.s., not significant.

We further examined cortical lamination, neuronal production, and apoptosis in P10 control and *Nex-Cre-Dicer Ko* brains. Numbers of Tbr1^+^ and Ctip2^+^ early-born neurons were increased and decreased in *Dicer Ko* cortices, respectively, compared to controls (**Figures 3A,B**). Conversely, quantification of Satb2^+^ and Cux1^+^ late-born neurons revealed no significant difference between control and *Dicer Ko* cortices (**Figures 3A,B**). Subsequently, we analyzed neuron and cell number by NeuN and DAPI immunostaining in the P10 cortex. Interestingly, countings of NeuN^+^ and DAPI^+^ cells within fixed columns of the cortical wall revealed no significant alterations in neuronal or cell number in *Nex-Cre-Dicer Ko* cortices, despite its significantly thinner cortex (**Figures 3C,D**). Further TUNEL analysis in the P10 cortex revealed no significant differences in the numbers of apoptotic cells in *Dicer Ko* and control brains (**Figures 3E,F**).

Our results indicate that even though the numbers of early- and late-born neurons, and apoptotic cells show temporal changes in postnatal cortices of *Nex-Cre-Dicer Ko* mice, overall cortical lamination and neuronal production remain undisrupted.

### CONDITIONAL *Dicer* ABLATION AFFECTS NEURON AND CELL PACKING DENSITY WITHIN THE CORTEX

**FIGURE 4:**
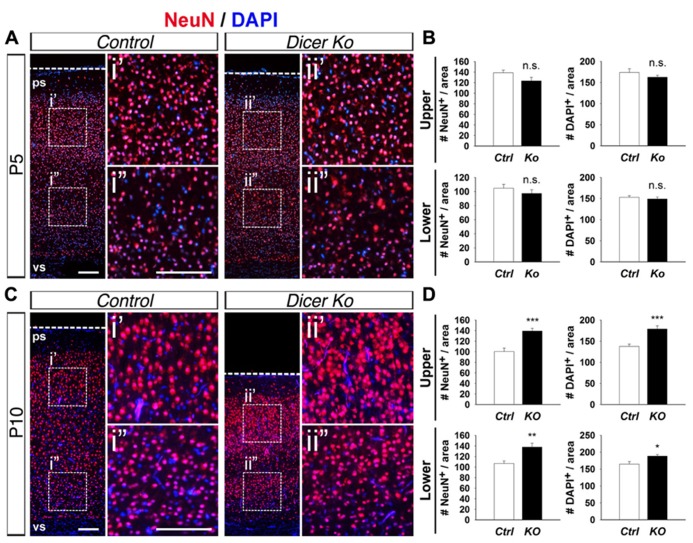
**Conditional Dicer ablation increases neuron and cell packing density within the cortex at P10.** **(A,B)** Numbers of NeuN^+^ and DAPI^+^ cells were unaffected in the upper and lower regions of the cortical wall of P5 *Nex-Cre-Dicer Ko* (*Ko*) cortices compared to controls (*Ctrl*). **(C,D)** Numbers of NeuN^+^ and DAPI^+^ cells in the upper and lower regions of the cortical wall were significantly increased in P10 *Dicer Ko* cortices compared to controls. The dashed boxes indicate the region shown at higher magnification in panel i′, i^′′^, ii′, ii^′′^; the boxed area in this region was chosen for subsequent analysis. The ventricular surface (vs) and pial surface (ps) are labeled. Scale bar: 100 μm. Data are presented as mean ± SEM; *n* ≥ 3 in all genotypes; *p* values in relation to control (*p < 0.02, **p < 0.004, ***p < 0.0008). n.s., not significant.

Considering that inactivation of Dicer in PNs did not adversely affect cortical lamination and neuronal production and only had a transient effect on cell survival, we investigated the cause of the smaller cortex in *Nex-Cre-Dicer Ko* mice further. We analyzed the density of neurons and cells by quantifying the number of NeuN^+^ and DAPI^+^ cells within uniform boxed areas in the upper and lower regions of the CP. At P5, there were no alterations in NeuN^+^ neuron and DAPI^+^ cell density in *Dicer Ko* cortices compared to controls (**Figures 4A,B**). However, P10 *Dicer Ko* cortices revealed significantly increased NeuN^+^ and DAPI^+^ cell numbers compared to controls, indicating increased density and packing of cells within the cortex during the stage of neuronal maturation (**Figures 4C,D**). These results demonstrate that Dicer ablation in PNs does not cause defective neuronal production but alters the neuronal packing density within the cortex.

### LOSS OF *Dicer* CAUSES ABNORMAL NEURONAL MATURATION WITH SHORTER NEURITE OUTGROWTH AND SMALLER CELL BODY SIZE

**FIGURE 5:**
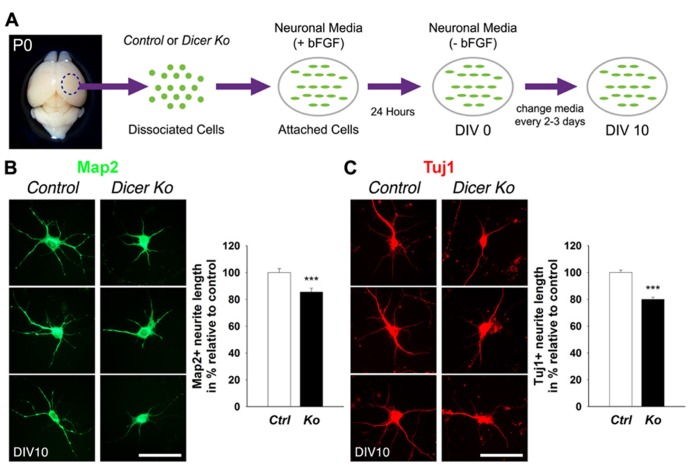
**Loss of Dicer in mature neurons delays neurite outgrowth *in vitro*.** **(A)** An illustrative summary of primary neuronal culture derived from control (*Ctrl*) and *Nex-Cre-Dicer Ko* (*Ko*) P0 dorsal cortex. **(B)** Measurements of Map2^+^ processes revealed shorter neurite outgrowth 10 days *in vitro* (DIV 10) in *Dicer Ko* (*N* = 113) neural cultures compared to controls (*N* = 158). **(C)** Measurements of Tuj1^+^ processes displayed shorter neurite outgrowth in DIV 10 *Dicer Ko* (*N* = 411) neural cultures compared to controls (*N* = 423). Scale bar: 50 μm. Data are presented as mean ± SEM; *n* ≥ 3 in all genotypes; *p* values in relation to control (***p < 0.0008).

**FIGURE 6:**
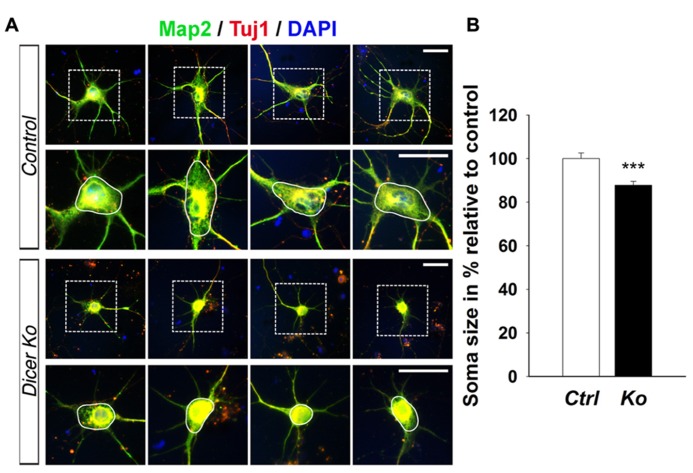
**miRNA depletion in maturing cortical neurons causes a reduction of soma size *in vitro*.** **(A)** Immunofluorescence microscopy of control (*Ctrl*) and *Nex-Cre-Dicer Ko* (*Ko*) primary neural cultures at 10 days *in vitro* (DIV 10) showing Map2 (green), Tuj1 (red), and DAPI (Jentarra et al., 2010). The dashed box indicates the region shown at higher magnification. The area encircled by the white line indicates the region of soma size analysis. **(B)** Measurements of soma size area in DIV 10 primary neurons revealed a significant reduction in *Dicer Ko* (*N* = 324) cell body size compared to controls (*N* = 259). Scale bar: 25 μm. Data are presented as mean ± SEM; *n* ≥ 3 in all genotypes; *p* values in relation to control (***p < 0.00009).

Given that the packing density of neurons was dramatically increased in the *Nex-Cre-Dicer Ko* cortices, we decided to further analyze neuronal morphology *in vitro*. This was done by harvesting cortical neurons from P0 mouse brains and culturing them under differentiation conditions using previously described methods with modifications (**Figure 5A**; Yu et al., 2005). After 10 days *in vitro* (DIV 10), cultures of differentiated neurons from control and *Dicer Ko* cortices were labeled with antibodies against Map2 and Tuj1 to illustrate neurites. We found that *Dicer Ko* neurons displayed significantly shorter neurites and processes compared to controls (**Figures 5B,C**). We next quantified soma size by measuring the cell body area of Map2- and Tuj1-stained neurons. Analysis of soma size revealed that *Nex-Cre-Dicer Ko* neurons displayed significantly smaller cell body area compared to controls (**Figures 6A,B**). Our results indicate that miRNA function is required for proper neurite outgrowth and soma size development of differentiated neurons during maturation.

## DISCUSSION

MiRNAs have been found to be crucial for proper development of the CNS. The results reported here underscore the importance of Dicer and miRNAs for neuronal differentiation and maturation. Although removal of Dicer in postmitotic cortical neurons has no immediate impact on neurogenesis, neuronal survival, or layer organization, it has dramatic effects on neurite outgrowth and cortical packing density. Consequently, Dicer-deficient mice exhibited thinner cortical walls and a progressive decline in postnatal growth, resulting in neurodegeneration defects. In conclusion, our results provide evidence that Dicer and miRNAs function is essential for neuronal maturation and that interference with the miRNA pathway results in phenotypes similar to neurodegenerative diseases.

Previous studies have revealed essential roles of miRNAs for neural progenitor proliferation, survival, and differentiation through Dicer ablation during embryonic development of the mouse neocortex (De Pietri Tonelli et al., 2008; Kawase-Koga et al., 2009, 2010; Andersson et al., 2010; Nowakowski et al., 2011). Moreover, limited studies have examined the role of Dicer in specific subpopulations of neurons, such as Purkinje cells, dopaminergic neurons, and excitatory neurons (Kim et al., 2007; Schaefer et al., 2007; Davis et al., 2008). In our mouse model, Dicer is ablated in PNs with the *Nex-Cre* line. Although the *Nex-Cre* line displays activity in the cortex by E13.5 (Goebbels et al., 2006), our model system reveals no significant alterations in brain weight or morphology in *Dicer* deficient mice at P1 (data not shown). This is perhaps caused by a delayed Dicer deletion, which allows a low level of Dicer proteins to continue to process miRNAs and regulate PNs until complete inactivation (Harfe et al., 2005; Kawase-Koga et al., 2009). MiRNAs are expressed in a diverse spectrum and change dynamically during brain development (Lagos-Quintana et al., 2002; Krichevsky et al., 2003; Miska et al., 2004; Sempere et al., 2004; Smirnova et al., 2005). Moreover, conserved complex interactions of multiple genes form a wide regulatory network in the developing cortex (Guillemot, 2005; Molyneaux et al., 2007). As such, the slight alterations in early- and late-born neuron populations in P5 and P10 *Nex-Cre-Dicer Ko* cortices are perhaps a balanced outcome of a multitude of distinct miRNAs with a variety of regulatory functions and targets.

Given the significant reduction in postnatal cortical growth in *Nex-Cre-Dicer Ko* brains, it is surprising to find preservation of neuronal cell numbers in the cortex. Moreover, despite a temporal increase of apoptotic cells in P5 cortices, *Nex-Cre-Dicer Ko* mice do not exhibit significant cell death. These results are in direct contrast to previous studies of Dicer function in Purkinje neurons and DAT-expressing neurons, which found widespread and continuous neurodegeneration and neuronal cell death (Kim et al., 2007; Schaefer et al., 2007). Moreover, compared to Dicer ablation studies in embryonic neural progenitors, which found dramatic apoptotic and differentiation defects, our studies have shown that loss of Dicer activity in postmitotic cortical neurons has minimal impact on neuronal survival (De Pietri Tonelli et al., 2008; Kawase-Koga et al., 2009). This mild apoptosis defect is similar to observations in *Dicer Ko* mice generated using the *CaMKII-Cre* line (Davis et al., 2008). These results highlight the diverse and variable functions Dicer and miRNAs carry for cell survival of different cell types at different time points during development.

Although blocking miRNA biogenesis in mature neurons reveals no apparent loss of neurons in the cortex, we have found a major increase in neuronal density in the cerebral cortex. This indicates that neuronal cell volume rather than neuron number is altered by depletion of Dicer and miRNAs in postmitotic cortical neurons. Moreover, direct differentiation of PNs from *Dicer* deficient cortices in a cell culture system has shown defects in neurite outgrowth (dendrites and axons) and decreased soma size. Decreased neurite outgrowth and increased packing density may contribute to reduced brain size in our *Nex-Cre-Dicer Ko* mice and in *Dicer Ko* mice generated using the *CaMKII-Cre* line (Davis et al., 2008). Moreover, our findings further support previous work, which have found a causal link between specific miRNAs such as miR-134, miR-34, miR-124, miR-9, and miR-132 with neurite outgrowth and elaboration *in vitro* (Vo et al., 2005; Yu et al., 2008; Agostini et al., 2011; Gaughwin et al., 2011; Clovis et al., 2012; Franke et al., 2012).

In conclusion, our results shed light on the essential role of Dicer-mediated miRNA functions for postmitotic neuronal maturation. Although loss of miRNA function in postmitotic cortical neurons has no definitive impact on neurogenesis, cortical patterning, or cell survival, it causes an atrophic change in neurites (dendrites and axons) and soma size. The aforementioned neurite outgrowth phenotypes are comparable with mouse models of neurodegeneration, which induce generalized atrophy of neuronal soma, dendrites and axons in the brain (Sakai et al., 2006). Increased packing density is also detected in a mouse model of Rett syndrome/X-linked mental retardation (Jentarra et al., 2010). Moreover, abnormally high packing density has been observed in patient brains with Rett syndrome, Williams syndrome, and schizophrenia (Bauman et al., 1995; Selemon et al., 1995; Galaburda et al., 2002). Our model of mature neuron degeneration bears resemblance to cell pathologies associated with schizophrenia and neurodegenerative diseases. As such, understanding the role of specific miRNAs during processes such as neuronal differentiation and maturation may be fundamental to discovering the morphological mechanisms of neurological disorders.

## Conflict of Interest Statement

The authors declare that the research was conducted in the absence of any commercial or financial relationships that could be construed as a potential conflict of interest.
